# Sarcopenia-related features and factors associated with lower muscle strength and physical performance in older Chinese: a cross sectional study

**DOI:** 10.1186/s12877-016-0220-7

**Published:** 2016-02-15

**Authors:** Ping Zeng, Yiwen Han, Jing Pang, Sinan Wu, Huan Gong, Jianguo Zhu, Jian Li, Tiemei Zhang

**Affiliations:** The Key Laboratory of Geriatrics, Beijing Hospital & Beijing Institute of Geriatrics, Ministry of Health, Beijing, 100730 China

**Keywords:** Sarcopenia, Muscle mass, Muscle strength, Physical performance

## Abstract

**Background:**

The associations of sarcopenia with adverse health status have highlighted the importance of sarcopenia research and intervention. This study was designed to analyze the characteristics of aging-related differences in appendicular skeletal muscle mass (ASM), handgrip strength (HS), gait speed (GS) and their associated factors in older Chinese, in order to generate guidance for sarcopenia intervention in this population.

**Methods:**

Population-based cross-sectional study. The criteria proposed by Asian Working Group for Sarcopenia were used to define low ASM, HS, and GS. The time required for five repeated chair stands (RCS) was also measured to evaluate physical performance. The differences of continuous variables were compared using one-way ANOVA tests and the Pearson correlation was used to analyze the relationship of each measurement adjusted by gender and age. Stepwise logistic regression was used to determine associated factors of low HS and low physical performance.

**Results:**

The data were analyzed in a total of 218 younger adults (aged 20–59, 76 males, 142 females) and 461 older adults (≥60 year, 207 males and 254 females). There were significant differences among age groups for HS, GS, and RCS while females were found to have significantly lower HS and GS values. ASM was significantly correlated with HS but not with other measures. Correlations among HS and GS, RCS were influenced by age differences. In the older group, unstructured daily routine (OR = 2.77) was associated with the risk of low GS, while physical exercise (OR = 0.27), and engaging in hobbies (OR = 0.11) were associated with faster GS. Co-morbidity (OR = 1.99) was associated with the risk of reduced performance of RCS, while engaging in hobbies was associated with faster RCS performance (OR = 0.35).

**Conclusions:**

Muscle strength and physical performance varied with aging in older Chinese. Measures of GS, HS, and RCS provide a readily available and effective method for assessing the risk of functional mobility decline. Maintaining a healthy life style and physical activity throughout life is beneficial for older people to improve their physical performance, especially in the early stages of aging.

## Background

Sarcopenia has been widely recognized as a syndrome featured by age-related loss of skeletal muscle mass, decrease of muscle strength and/or physical performance in older populations [1, 2]. Because of the associations of sarcopenia with adverse health status [3–8], it has become a subject of increased focus in geriatrics research. In 2014, the Asian Working Group for Sarcopenia (AWGS) has proposed a consensus for the diagnosis of sarcopenia [9]. Taking an approach similar to that of the European Working Group on Sarcopenia in Older Peoples (EWGSOP) [10], diagnostic criteria for three parameters (muscle mass, muscle strength, and physical performance) were determined. However, ethnic diversity in these measurements has been found [11–14], even within Asia [14]. Furthermore, the prevalence of reduced muscle mass reported in Asian populations is extremely low [15–17], while it is a pre-requisite for the diagnosing of sarcopenia. Except for identifying individuals at especially high risk of sarcopenia, the exact definition of sarcopenia may not be as important as developing criteria for intervention to maintain the function of older people. Some studies have confirmed the potential benefits of sarcopenia intervention approaches [18–21] in reversing age-related declines in muscle mass and physical performance while increasing in mortality, which support the importance of further understanding the sarcopenia-related factors in national populations, especially in China that has a large size of rapidly aging population and a huge number of disabled older people. In turn, this information can be used to formulate and implement strategies for sarcopenia intervention and prevention. In this paper, the age-related differences in muscle mass, muscle strength, and physical performance associated with sarcopenia are described. The correlations between each variable and the various factors associated with the parameters of sarcopenia are examined quantitatively.

## Methods

### Study design

This investigation used a cross-sectional study design. In response to the need for understanding the situation of sarcopenia in China, we collected data to examine age-related variations of body muscle mass, muscle strength, and physical performance in two study and one reference groups. The survey was conducted during 2013 in two groups of older volunteers (≥60 year), who were retired office workers living in the urban area of Beijing and older farmers living in Jingzhuang, Yanqing, 80 kilometer northwest of Beijing. In addition, young adult (20–59 years) employees of Beijing Hospital also volunteered to participate to make up the young reference group. Data were gathered from 465 older participants and 220 younger adults. Due to incomplete data collection or artificial implants, such as cardiac pacemakers or joints which hinder the subjects completing the bioimpedance measurement of body composition, the data were only analyzed for 679 subjects (76 young males, 142 young females, 207 older males and 254 older females).

Questionnaires and physical examinations were used to collect information on demographics, life style, history of diseases, physical status, activities of daily living (ADL), skeletal muscle mass, muscle strength, and physical performance. The life style variables included: current smoking (≥5/day); current alcohol consumption (at least once a week); engaging in physical exercise (moderate or more, at least once a week and lasting for 30 min or more); meals per day; daily routine (daily time schedule, 1 = structured,2 = moderate,3 = unstructured); and having hobbies (playing chess, reading, drawing, painting, handwriting, singing, planting, fishing, keeping pets). Meals per day was investigated in this study as a surrogate of nutrition intake. Daily routine and hobbies were considered as they may indicate the psychological status and life attitude of older people. Individuals with 2 or more common diseases, such as hypertension, diabetes, cardiovascular diseases, coronary heart disease, chronic obstructive pulmonary disease (COPD), osteoarthritis, and cancer, were considered as having co-morbidity. All the participants were ambulatory without major physical disabilities. The interviewers were trained before the survey was administered; the subjects’ informed consent was obtained to use their information in this study; and the study was approved by the Ethics Committee of Beijing Hospital, Ministry of Health.

### Body composition measurements

Subjects were dressed in light clothes and were barefoot. Weight and height were measured using a calibrated digital scale. Weights were rounded to the nearest 0.1 kg, and heights were rounded to the nearest 0.1 cm. Body mass index (BMI) was calculated by dividing the weight by the square of the height (kg/m^2^).

Body composition was measured with a body composition analyzer (BCA-2A, Tongfang Health Technology Company, Tshinghua, Beijing, China), which conducted a direct segmental multi-frequency (5 kHz to 500 kHz) bioelectrical impedance analysis (BIA). The details of measurement of body mass by BCA-2A have been published elsewhere [22]. In short, the BCA-2A has eight tactile electrodes allowing the subjects’ hands and feet to be separately measured for bioelectrical resistance. While being measured, the participants stood on the device in bare feet with legs slightly apart while holding the electrodes in both hands with arms away from the trunk. After the measurement, the analyzer generated skeletal muscle mass (SM), appendicular skeletal muscle mass (ASM), lean mass (LM), and fat mass (FM) reports on the screen. The measurements were calibrated using a patented formula obtained by magnetic resonance imaging (MRI) analysis from a group of healthy Chinese subjects. The ASM index (ASMI), SM index (SMI), and LM index (LMI) were obtained by dividing ASM, SM, and LM by the square of height, respectively (kg/m^2^).

### Muscle strength and physical performance measurements

Muscle strength was determined by handgrip strength (HS) and was measured in each hand using a hand dynamometer (WCS-II, Beijing). The grip strength was measured twice for each hand, and the higher of the two values was used in the analysis.

Two physical performance measures were considered in this study: usual gait speed (GS) and five times repeated chair stands (RCS). GS was evaluated by the 6-m gait speed (GS) test. Participants walked at their usual pace from a standing start and continued walking to a point past the line indicating the end of the course. The total time—from the “start” command issued by the investigator to the first step over the end line—was measured with a stopwatch. GS was calculated by dividing distance in meters by the time in seconds (m/s). When measuring the time for RCS, participants were asked to rise from a sitting position and stand up completely as quickly as possible, repeating this movement five times in rapid succession, without using their arms. The exercise was stopped if there were concerns about the subject’s safety or if the procedure could not be completed within 1 min.

### Definition of low muscle mass, muscle strength and physical performance

The criteria proposed by the Asian Working Group for Sarcopenia (AWGS) were used to define sarcopenia related parameters, i.e. low muscle mass, low muscle strength, and low physical performance (defined by low GS). Reduced physical performance of RCSs means a longer time to complete the RCSs. Since the cut-off value to define reduced performance of RCS was not available in AWGS, the approach of previous work for generating a reference value was used [11, 23]. That is, a reduced performance of RCS was defined as a value in excess of the 80th percentile of RCSs measured in the older participants in this study. As a result, RCS ≥12.5 s was defined as reduced RCS performance.

### Statistical analysis

Descriptive statistics were used to characterize the demographics and measured variables of the subjects. The differences of continuous variables between younger and older adults were compared using one-way ANOVA tests. To analyze the degrees of differences in ASMI, GS, HS, and RCS among age groups, the mean values (X_n_) of the variables obtained in the groups aged ≥ 50 (50–59, 60–69, 70–79, 80+) were compared with those (X) obtained in the younger adults (20–49 years), and the degrees of difference were calculated as 100*(X_n_-X)/X (Comparison 1). The mean values (X_n_) of these variables obtained in the groups aged ≥ 50 (50–59, 60–69, 70–79, 80-) were also compared with those of the next younger group X_n-1_ (group 50–59 was compared with group 20–49), and the degrees of difference were calculated as 100*(X_n_-X_n-1_)/X_n-1_ (Comparison 2). Pearson correlation was used to analyze the relationship of each measurement adjusted by gender and age. Stepwise logistic regression was used to determine associated factors of low HS and low physical performance. The statistical analyses were carried out using SAS software licensed to the Chinese Center for Disease Control and Prevention, and *p* <0.05 was considered as statistically significant.

## Results

### Descriptions of anthropometry, muscle strength and physical performance data between young and older groups

The data concerning anthropometry, muscle strength, and physical performance of young group and older group are shown in Table 1. Older people had significant lower LM, ASM, HS, but higher fat mass, and slower GS and RCS than young people (p < 0.0001). Males had significant higher LM, ASM, HS, and quicker GS, but lower FM than females (p < 0.05). The gender difference for RCS was not significant.

**Table 1:** The descriptions of anthropometry, muscle strength and physical performance data of young group and elderly group (mean ± SD)

Characteristic	Young group (20–59)		Older group (≥60)			
	Males (n = 76)	Female (n = 142)	Male (n = 207)	Female (n = 254)	p1	p2
Average age (year)	38.6 ± 12.0	41.7 ± 12.6	71.0 ± 5.7	69.2 ± 6.5	<0.0001	0.6297
Anthropometric measurements						
Height (m)	1.73 ± 0.07	1.61 ± 0.05	1.67 ± 0.06	1.56 ± 0.06	<0.0001	<0.0001
Weight (kg)	72.76 ± 11.22	59.55 ± 8.90	66.93 ± 10.00	60.05 ± 9.97	0.0221	<0.0001
Fat mass (kg)	14.84 ± 4.99	17.61 ± 5.60	16.48 ± 5.47	20.57 ± 5.97	<0.0001	<0.0001
Lean mass (kg)	54.43 ± 6.82	39.13 ± 4.10	47.63 ± 5.43	37.39 ± 4.88	<0.0001	<0.0001
Arm skeletal mass (kg)	7.90 ± 1.08	5.84 ± 2.52	6.88 ± 0.81	5.40 ± 0.74	<0.0001	<0.0001
Leg skeletal mass (kg)	19.35 ± 2.43	13.90 ± 1.46	16.96 ± 1.95	13.30 ± 1.73	<0.0001	<0.0001
ASM (kg)	27.25 ± 3.44	19.46 ± 2.06	23.84 ± 2.75	18.70 ± 2.42	<0.0001	<0.0001
Fat mass (%)	19.90 ± 4.04	28.98 ± 5.42	24.17 ± 5.56	33.73 ± 5.73	<0.0001	<0.0001
Lean mass (%)	75.00 ± 3.76	66.25 ± 5.12	71.66 ± 5.89	62.82 ± 5.73	<0.0001	<0.0001
Arm skeletal mass (%)	10.92 ± 0.96	9.91 ± 4.64	10.35 ± 0.90	9.08 ± 1.00	0.0001	<0.0001
Leg skeletal mass (%)	26.72 ± 1.32	23.54 ± 1.82	25.52 ± 2.09	22.35 ± 2.03	<0.0001	<0.0001
Percentage of ASM (%)	37.55 ± 2.12	32.93 ± 2.38	35.87 ± 2.98	31.42 ± 2.93	<0.0001	<0.0001
BMI (kg/m^2^)	24.09 ± 3.12	22.87 ± 3.30	23.88 ± 3.13	24.59 ± 3.71	0.0005	0.6346
LMI (kg/m^2^)	17.97 ± 1.62	15.02 ± 1.30	16.99 ± 1.45	15.29 ± 1.56	0.1064	<0.0001
Arm skeletal mass index (kg/m^2^)	2.62 ± 0.32	2.24 ± 0.98	2.46 ± 0.23	2.21 ± 0.24	0.0357	<0.0001
Leg skeletal mass index (kg/m^2^)	6.42 ± 0.61	5.34 ± 0.46	6.05 ± 0.52	5.44 ± 0.56	0.0755	<0.0001
ASMI(kg/m^2^)	9.00 ± 0.85	7.47 ± 0.65	8.50 ± 0.74	7.65 ± 0.78	0.2292	<0.0001
Muscle strength and body physical function						
Handgrip strength (kg)	40.81 ± 5.77	26.44 ± 4.42	34.04 ± 8.05	21.56 ± 5.64	<0.0001	<0.0001
Gait Speed (m/s)	1.51 ± 0.24	1.45 ± 0.22	1.19 ± 0.26	1.15 ± 0.31	<0.0001	0.0200
Chair repeat time (s)	6.58 ± 2.01	6.66 ± 4.27	10.66 ± 5.46	11.42 ± 7.41	<0.0001	0.2287

p1: Age difference; p2: gender difference (across full sample)

### Degrees of difference in muscle mass, muscle strength, and physical performance by age and gender

The degrees of difference in ASMI, GS, HS and RCS with age are shown in Table 2. Declines in ASMI, GS, and HS were observed after years of 60s. When the mean values of the measured variables obtained in the groups aged ≥ 50 (50–59, 60–69, 70–79, 80+) were compared with those of younger adults (20–49, Comparison 1), GS declined more in females (17.24–40.69 %) than in males (18.00–28.67 %), while HS declined more in males (9.56–34.58 %) than in females (16.70–28.54 %). The time required to complete RCS increased more dramatically and earlier (at 50) than did other variables, with 17.49–84.90 % and 40.96–197.76 % increases in male and females, respectively. When the mean values of the measured variables obtained in the groups aged ≥ 50 (50–59, 60–69, 70–79, 80-) were compared with those of the next younger group (Comparison 2), two obvious declines in each variable (RCS increase) were observed in 60- and 80-year old group (Table 2, Comparison 2). The decline of ASMI was less significant than other variables in both males and females.

**Table 2:** The degrees of difference in sarcopenia related parameters with age

		ASM index((kg/m^2^)			Gait speed (m/s)			Handgrip strength (kg)			Repeated chair stands (s)		
Males	n	Mean ± SD	Comparison1 (%)	Comparison2 (%)	Mean ± SD	Comparison1 (%)	Comparison2 (%)	Mean ± SD	Comparison1 (%)	Comparison2 (%)	Mean ± SD	Comparison1 (%)	Comparison 2 (%)
20–49	56	8.88 ± 0.84	-	-	1.50 ± 0.24	-	-	40.6 ± 5.8	-	-	6.29 ± 2.0	-	-
50–59	20	9.31 ± 0.84	4.84	4.84	1.53 ± 0.25	2.00	2.00	41.53 ± 5.73	2.29	2.29	7.39 ± 1.84	17.49	17.49
60–69	82	8.55 ± 0.73	−3.72	−8.16	1.23 ± 0.28	−18.00	−19.61	36.72 ± 8.26	−9.56	−11.58	10.63 ± 7.62	69.00	43.84
70–79	112	8.50 ± 0.68	−4.28	−0.58	1.18 ± 0.25	−21.33	−4.07	33.37 ± 7.45	−17.81	−9.12	10.46 ± 2.59	66.30	−1.60
80-	13	8.26 ± 1.23	−6.98	−2.82	1.07 ± 0.29	−28.67	−9.32	26.56 ± 5.89	−34.58	−20.41	11.63 ± 3.65	84.90	11.19
Females													
20–49	92	7.31 ± 0.53	-	-	1.45 ± 0.19	-	-	26.1 ± 3.9	-	-	5.81 ± 1.40	-	-
50–59	50	7.69 ± 0.84	5.20	5.20	1.46 ± 0.28	0.69		26.95 ± 5.13	3.26	3.26	8.19 ± 6.63	40.96	40.96
60–69	146	7.69 ± 0.78	5.20	0.00	1.20 ± 0.29	−17.24	−17.81	21.74 ± 5.62	−16.70	−19.33	10.17 ± 3.32	75.04	24.18
70–79	86	7.66 ± 0.78	4.79	−0.39	1.13 ± 0.30	−22.07	−5.83	21.54 ± 5.59	−17.47	−0.92	11.82 ± 7.76	103.44	16.22
80-	22	7.14 ± 0.68	−2.33	−6.79	0.86 ± 0.22	−40.69	−23.89	18.65 ± 5.12	−28.54	−13.42	17.30 ± 16.46	197.76	46.36

Comparison 1, compare the mean values (X_n_) of the variable obtained in the groups aged ≥ 50 with those (X) obtained in the 20–49 years group, and calculated as 100*(X_n_-X)/X; Comparison 2: compare the mean values (X_n_) of the variable obtained in the groups aged 50 with the mean values X_n-1_of the next younger group, and calculated as 100*(X_n_-X_n-1_)/X_n-1_

### Correlation among appendicular muscle mass, appendicular muscle mass index, muscle strength, and physical performance

The Pearson correlation coefficients among ASM, ASMI, HS, GS, and RCS are shown in Table 3. In the older groups, ASM associated with HS (*r* = 0.28 in males, *p* < 0.001; *r* = 0.30 in females, *p* < 0.001); GS correlated with HS (*r* = 0.35 in males, *p* < 0.001; r = 0.40 in females, *p* < 0.001) and RCS (*r* = 0.26 in males, *p* < 0.001; *r* = 0.23 in females, *p* < 0.001). In the adults (age < 60), only appendicular muscle mass and HS were significantly associated (for ASM and HS, *r* = 0.24 in males, *p* < 0.001; *r* = 0.35 in females, *p* < 0.001). The correlations of ASMI with other variables were very weak in both older and adults groups.

**Table 3:** The correlation coefficient among appendicular muscle mass, appendicular muscle mass index, mass strength, gait speed, and repeated chair stands (r)

	≥60 years			<60 years		
Male	HS	GS	RCS	HS	GS	RCS
ASM (kg)	0.28*	0.02	0.06	0.24**	0.01	0.01
ASM index	0.05	−0.07	0.07	0.25**	−0.02	0.05
HS		0.35*	−0.15**		0.17	0.04
RCS	−0.14**	−0.26*		0.04	0.08	
Female						
ASM (kg)	0.30*	0.05	−0.02	0.35	0.05	−0.08
ASM index	0.07	−0.11	0.03	0.30*	−0.09	0.04
HS		0.40*	−0.17*		0.09	−0.08
RCS	−0.17*	−0.23*		−0.08	−0.12	

*: p < 0.01; **: p < 0.05. *ASM* appendicular muscle mass, *HS* handgrip strength, *GS* gait speed, *RCS* repeated chair stands

### Factors associated with low muscle strength and reduced physical performance in the older group

Factors, such as age, gender, lifestyle, and health status associated with low muscle strength, reduced physical performance are shown in Table 4. In general, aging was associated with the risk of low muscle strength and reduced physical performance, and rural farmers were weaker in muscle strength and physical performance. BMI and unstructured daily routine were associated with the risk of low GS, with OR (95 % CI): 1.25 (1.09–1.43) and 2.77(1.34–5.72), respectively, while physical exercise was associated with faster GS, having OR (95 % CI): 0.27 (0.09–0.79). Engagement in hobbies was also associated with better performance in both GS and RCS, OR (95 % CI): 0.11(0.04–0.33) and 0.35(0.17–0.72), respectively. In addition, co-morbidity was associated with the risk of reduced performance of RCS, OR (95 % CI): 1.99(1.20–3.29).

**Table 4:** Factors associated with low handgrip strength, low gait speed and reduced performance in repeat chair stands (RCS) by stepwise logistic regression (OR: 95 % CI)

Variables	Low handgrip strength	Low gait speed	Reduced performance of RCS
Age increased 10 years	1.13(1.07–1.18)	1.12(1.04–1.18)	1.10(1.06–1.15)
Urban resident	0.10(0.05–0.20)	0.08(0.02–0.40)	0.39(0.24–0.65)
Female	1.92(1.06–3.46)	-	-
BMI	-	1.25(1.09–1.43)	-
≥2 co-morbidity	-	-	1.99(1.20–3.29)
Current smoker	-	-	-
Current Drinker	-	-	-
Physical exercise	-	0.27(0.09–0.79)	-
Meals per day	-	-	-
Structured daily routine	-	2.77(1.34–5.72))	-
Hobby loving	-	0.11(0.04–0.33)	0.35(0.17–0.72)
Self rated health	-		-

Current smoker: ≥5/day; Current drinker: at least once a week; Physical exercise: moderate or more, at least once a week and lasting for 30 min or more; Meals/day: 1 = 1meal, 2 = 2meals, 3 ≥ 3meals; Structured daily routine: 1 = structured, 2 = moderate, 3 = unstructured; Self rated health: 1 = very bad, 2 = bad, 3 = modest, 4 = good, 5 = very good

## Discussion

In this study, patterns of differences in muscle mass, muscle strength, and physical performance over time, the correlation among these parameters, and other associated factors were analyzed in a Chinese population. The most obvious changes were observed in muscle strength and physical performance in the age 60–69 and 80+ groups. Additionally, muscle strength and physical performance (indicated by GS) showed significant gender differences, with females having poorer muscle strength and lower GS (Table 2). Our data imply that before the age of 60, there could be opportunities to intervene and prevent the decline of muscle mass or muscle physical function, and that older women should have greater concern about functional declines in their later years than men. An earlier study reported that a noticeable decrease in absolute skeletal muscle mass did not occur until the end of fifth decade life, which was attributed to a decrease in a lower body SM [24]. Our data show a very dramatic decline in RCS, which may represent the decline of leg muscle strength, supporting the idea that an important benefit may be gained by maintaining muscle mass and strength in the lower extremities.

Studies in China [25] and Asia [15, 16] have found a low prevalence of low ASM, especially among older women. In our previous report [22], several factors which may cause bias in the determination of the reference value of for ASM were discussed to explain this phenomenon. In this study, the prevalence of low ASM was only 1.44 % in males and 0.39 % in females. Since low ASM is a pre-requisite for the definition of sarcopenia, the prevalence of sarcopenia is expected to be low in these populations. Our results demonstrated that muscle strength and physical performance declined to a greater extent than did muscle mass, which is consistent with previous reports (Table 2) [26].

Statistical correlations among the sarcopenia parameters (e.g., ASM, HS, GS, and RCS) were analyzed to investigate associations among these factors. In our study, ASM was only correlated with HS (both in adult and older groups; Table 3), not with physical performance. Similar results were also reported in a Taiwanese population [13]. The correlations among muscle strength and physical performance were shown to vary by age category. In the adults, these correlations were not statistically significant (p > 0.05), while in the older group, GS was statistically correlated with HS and RCS. The age specific association of muscle function supports sarcopenia being described as part of the geriatric syndrome [27]. Some reports have demonstrated that HS and GS are associated with adverse health-related events in the older population [4, 28–30]. The poor association between muscle mass and physical performance, and low prevalence of reduced muscle mass may indicate that, even without muscle mass measurement (which is still challenged by precision and proper definition), the measurement of muscle strength and physical performance would be good predictors of future functional status in the older population. These parameters are also easily obtained in screening or clinical tests to evaluate the muscle strength and physical performance in older people.

Using the AWGS criteria, 5.31 % of males, and 7.50 % of females had low GS; 13.10 % of males and 22.86 % of females had low HS in our older population. Although RCS was not a parameter proposed by AWGS for sarcopenia screening, other researchers have found RCS to be a sensitive indicator of the strength of the lower limbs [29] and is, therefore, included in our list of parameters. Our study found that the time to complete five RCS increased more dramatically and earlier (at 50s) than other variables, with 17.5–84.9 % and 41.0–197.8 % increases in male and females, respectively. Using the approach of previous work for generating reference values [11, 23], a value in the excess 80th percentile of RCSs (≥12.5 s.) measured in the older people of this study was defined as the criterion for indicating reduced RCS performance. Using this criterion, 18.36 % of older males and 27.60 % of older females presented reduced RCS performance. Another study defined a cut-point of 17.05 s and showed that it was an adequate substitute to predict adverse health events [29]. In other studies, Caucasians were shown to have longer RCS than Asians [14, 31]. The ethnic differences in RCS may reflect the higher BMI and body weights of Caucasians.

Lifestyle factors, such as nutrition, physical activity, exercise, alcohol intake, and tobacco use, have an impact on the occurrence of sarcopenia [32–34]. Our study demonstrated that physical performances are more likely to be correlated with lifestyle factors, while HS is not. Greater strength may be more associated with a better nutrition, especially nutrition experienced in the early stages of life or even during fetal development [35, 36]. This observation leads to the special concerns about the nutrition status of older Chinese, since nutrition is associated with muscle mass, and our population has a relatively higher prevalence of reduced HS (13.10 % in males and 22.86 % in females). Obesity and unstructured daily schedule were associated with the risk of low GS, while physical exercise is associated with faster GS. Engagement in hobbies was associated with better performance in both GS and RCS. These results may indicate that a positive and healthy life style contributes to improved physical performance of the older people [37]. In addition, among three parameters; i.e., HS, GS, and RCS, co-morbidity was the only factor associated with the risk of reduced performance of RCS. Whether RCS contains more information about the health status in the older population needs further study, our investigation supports the importance of measuring RCS to evaluate lower extremity functions in the older people.

This study has several limitations. First, it is a cross-sectional study, which makes it less likely to observe the changes of muscle mass, muscle strength, and physical performance with aging. Specifically, the study group was relatively healthy volunteers, and the sample size was not sufficiently large; therefore, it is difficult to produce a representative description of sarcopenia in our population. However, at a time when related studies are still scarce, our study provides information that is helpful to form potential strategies for early intervention and prevention of sarcopenia in older Chinese. Also, due to the low prevalence of reduced muscle mass in this population, the specific factors associated with sarcopenia cannot be evaluated. However, potential factors that may influence muscle strength and physical performance have been identified and significant associations have been observed.

## Conclusions

Physical performance and muscle strength declined with age in an older Chinese population. Measures of physical performance and muscle strength are easily obtained and represent an effective way to assess the risk of mobility functional decline in the older people. Maintaining a healthful life style and physical activity throughout the life-course is beneficial for older people to improve their physical performance, especially in the early stage of aging.

## Abbreviations

ASM: appendicular skeletal muscle mass
ASMI: appendicular skeletal mass index
AWGS: the Asian Working Group for Sarcopenia
BIA: bioelectrical impedance analyzer
BMI: body mass index
EWGSOP: the European Working Group on Sarcopenia in Older Peoples
GS: gait speed
HS: handgrip strength
LM: lean mass
MRI: magnetic resonance imaging
RCS: repeated chair stands
SM: skeletal muscle mass
